# Economic cost of strategic implementation approaches to increase uptake of digital therapeutics for substance use disorders in a large integrated health system

**DOI:** 10.1371/journal.pdig.0001145

**Published:** 2026-01-08

**Authors:** Edwin S. Wong, Caitlin N. Dorsey, Tara C. Beatty, Jennifer F. Bobb, Kelsey Stefanik-Guizlo, Dustin L. Key, Arvind Ramaprasan, Abisola E. Idu, John C. Fortney, Jessica Mogk, Lorella Palazzo, Ryan M. Caldeiro, Deborah King, Angela Garza McWethy, Joseph E. Glass

**Affiliations:** 1 Department of Health Systems and Population Health, University of Washington, Seattle, Washington, United States of America; 2 Kaiser Permanente Washington Health Research Institute, Seattle, Washington, United States of America; 3 Center of Innovation for Veteran-Centered and Value-Driven Care, Veterans Affairs Puget Sound Health Care System, Seattle, Washington, United States of America; 4 Department of Psychiatry and Behavioral Sciences, University of Washington School of Medicine, Seattle, Washington, United States of America; Polytechnic Institute of Porto: Instituto Politecnico do Porto, PORTUGAL

## Abstract

Evidence-based digital therapeutics are a promising approach for the scale-up of substance use disorder (SUD) treatments. Despite demonstrated efficacy, utilization of digital therapeutics is low. Strategic implementation approaches have potential for increasing digital therapeutic use. Applicability to health systems depends, in part, on the economic costs. The objective of this study was to describe implementation and intervention costs of implementation strategies to increase uptake of an evidence-based digital treatment for SUD. We conducted an economic evaluation alongside a hybrid type III cluster-randomized trial within a large integrated health system. All clinics implemented a standard implementation (SI) strategy, and clinics were assigned using 2x2 factorial randomization to additionally receive practice facilitation (PF) and/or health coaching (HC). Implementation costs included the cost of time devoted to implementation activities and direct operating costs. Time devoted to implementation activities was ascertained through structured meeting logs and time use surveys. Operating costs were captured using project budget reports. Intervention costs included expenses for prescriptions and healthcare encounters related to the digital therapeutic, measured using electronic health record data. Univariate statistics were calculated for cost estimates with comparisons presented by trial arm, implementation activity, staff role and study month. Analyses were conducted from a health system perspective. Twenty-one primary care sites participated in the trial. Over the 50-month study period, the total cost of all implementation activities was $748,088. Implementation costs per clinic were highest in the SI + PF + HC arm ($48,029), followed by SI + HC ($36,544), SI + PF ($30,665) and SI alone ($24,774). Intervention costs were highest in the SI + PF + HC arm ($18,051), followed by SI + PF ($11,492), SI + HC ($967) and SI alone ($1,879). Findings from this study can guide health systems by informing the economic investment required to employ implementation strategies demonstrated to increase uptake of evidence-based practices for behavioral health conditions.

Trial Registration: NCT05160233.

## Author summary

Digital therapeutics are an efficacious approach to treating substance use disorder, however, uptake is low. Strategic implementation approaches to facilitate uptake of evidence-based practices have the potential for increasing use of digital therapeutics. Of the limited number of studies examining economic aspects of implementation approaches, none have quantified costs of implementing practices for substance use disorder. To our knowledge, this is the first study to quantify the costs of implementing an evidence-based practice for substance use disorder in a real-world setting. Findings from this study can inform decisions to adopt evidence-based approaches for substance use disorder into routine clinical workflows.

## Introduction

The US Preventive Services Task Force, World Health Organization and others recommend universal screening for unhealthy alcohol and drug use in primary care settings [1–4]. For those who are identified through screening as having a substance use disorder (SUD), recommended next steps for intervention include behavioral counseling, pharmacological interventions, and/or referral to specialty treatment.

Digital therapeutics represent a promising approach to scale up the treatment of SUDs and could be integrated into care pathways recommended by stakeholders. These therapeutics work by delivering treatment content including assessments, treatment modules, and normed feedback electronically through websites or smartphone apps, often under the supervision of a clinician [5]. For example, the prescription digital therapeutics reSET® and reSET-O® received U.S. Food and Drug Administration authorization for the treatment of SUD and opioid use disorder (OUD), respectively [6]. reSET and reSET-O incorporate three evidence-based cognitive-behavioral interventions: community reinforcement approach [7], contingency management [8] and fluency training [9]. The community reinforcement approach is delivered through more than 60 interactive modules in reSET and reSET-O, covering topics such as drug refusal skills, interpersonal communication strategies, problem solving techniques, exploration of the causes and consequences of substance use, and learning how to replace unhealthy habits with healthier alternatives. Each module is designed to take 20–30 minutes to complete. Fluency training reinforces learning by quizzing patients on newly learned material. The contingency management component incentivizes successful module and quiz completion, as well as clinician-reported negative urine drug screens. These incentives are delivered via online gift cards of randomly assigned monetary values. A prior study demonstrated reSET-O improved quality adjusted life years and reduced overall medical costs among patients with opioid use disorder, suggesting this therapeutic is a cost-effective approach for health care payers [10–12].

Digital therapeutics can be particularly valuable for primary care settings, which often serve as the center of a patient’s care management and the entry point for new care episodes. A recent systematic review revealed offering digital therapeutics in primary care can yield benefits to health systems, including reduced health expenditures, improved resource utilization, and enhanced workflow for health professionals [13]. Additionally, the National Academy of Medicine and other experts call for the prevention and treatment of SUDs in primary care [14–17]. Primary care practices are increasingly taking steps to recognize SUDs. Integrating treatment with identification of SUDs within the same setting can increase care continuity and reduce time to treatment. Yet, primary care staff currently lack treatment options [18]. For instance, buprenorphine, a lifesaving treatment for OUD, has limited implementation within primary care settings. Digital therapeutics hold strong potential for overcoming the barriers to treating SUDs in primary care, and can also complement buprenorphine treatment [19,20]. Several digital therapeutics have been shown efficacious in randomized control trials in increasing odds of abstinence and reducing dropout from treatment [19–21], and may reduce clinician time by 50–80% [20,22].

Despite demonstrated efficacy for treating SUDs and potential as a care option in primary care settings, uptake of digital therapeutics remains low. Prior research indicates about 20–25% of patients with SUD ever receive treatment [23,24], and few health organizations having implemented digital therapeutics for SUD [25]. Reasons for low uptake include barriers to utilization of care for SUD, both digital and non-digital modalities, such as patients’ failure to perceive a need for treatment and stigma around SUD care [26–29]. Also, limited access to digital therapeutics is due to lack of a consistent approach for reimbursement and payment, lack of provider awareness and hesitation to adopt stemming from the novelty of the treatment, absence of standard clinical protocols for prescribing and longitudinally monitoring digital treatments, and the absence of digital treatments in standard formularies [30,31].

Strategic implementation approaches could be beneficial in addressing these challenges, particularly within primary care settings that are part of integrated health systems. In this study, we examine the economic considerations of using several implementation strategies aimed at increasing provider adoption and patient reach of the digital therapeutics reSET and reSET-O. To our knowledge, this study is the first to examine the costs of strategies for implementing digital therapeutics and contributes to an emerging literature on the economic impacts of implementation.

The objective of this study was to investigate the implementation and clinical intervention costs stemming from strategic approaches to increase uptake of an evidence-based digital treatment for SUD. Costs were assessed as part of an implementation trial within a large integrated health system located in the Pacific Northwest.

## Materials and methods

### Overview of study design

We conducted an economic evaluation by examining the costs associated with implementing two similar evidence-based digital therapeutics for SUD that were provided by the same vendor. Economic evaluation involved prospective collection of implementation activity data alongside a hybrid type III effectiveness-implementation trial [32]. Collected data included information on both implementation and clinical intervention costs to assess the total cost to the healthcare system of adopting the digital therapeutics [33].

### Study setting and implementation trial

This economic analysis was conducted as part of a hybrid type III cluster-randomized trial within a large integrated health system in the Pacific Northwest headquartered in Seattle, WA (ClinicalTrials.gov Identifier: NCT05160233). Full details of the trial design are published elsewhere [34]. The trial sought to test the effectiveness of implementation strategies to increase patient reach and fidelity of use of the digital therapeutics to clinical recommendations. The therapeutics included reSET, an FDA-authorized digital therapeutic designed to augment care for SUD, and a similar therapeutic tailored for OUD, reSET-O [6,20,35,36]. To achieve this goal, 22 primary care clinics were randomized at 21 primary care sites (one small clinic was paired with a nearby large clinic to match how the two clinics operate). Sites were randomized to receive implementation strategies using a 2x2 factorial design, resulting in four implementation approaches: standard implementation only; standard implementation plus practice facilitation; standard implementation plus health coaching; and standard implementation, practice facilitation, and health coaching.

Standard implementation, a strategy previously used by the health system to facilitate adoption of smartphone apps, centered around training for integrated mental health (IMH) specialists (licensed social workers) provided by delivery system leaders and the digital therapeutics vendor. The training covered topics such as research and evidence behind the digital therapeutics and clinical procedures for use of the therapeutics in primary care. Standard implementation also included the development and utilization of resources such as a toolkit for primary care clinics with job aids, patient pamphlets, and scripts to help clinicians enroll patients, as well as electronic health record (EHR) tools to help clinicians document and prescribe reSET and reSET-O. The second strategy, standard implementation plus a clinician-facing practice facilitation approach, involved trained facilitators meeting with clinics to bolster education, audit and provide feedback, support Plan-Do-Study-Act cycles, and engage others in increasing use of the therapeutics [37–39]. The third strategy was standard implementation plus health coaching, where a credentialed medical assistant (MA) employed by the health care system conducted approximately weekly contact with patients using reSET or reSET-O, and outreach to patients prescribed but who were not engaging with the apps. The fourth strategy involved the combination of standard implementation, practice facilitation and health coaching.

The trial was conducted between December 2021 and February 2023. Enrollment of sites was staggered during December 2021 to July 2022. Once enrolled, 20 sites were involved in implementation activities for 12 months, and two sites that were delayed in starting the trial were involved in implementation for about six months. Collection of implementation activity and cost data took place during the trial period, in addition to a 34-month period prior to the start of the trial (February 2019 to November 2021). This pre-trial data collection tracked implementation activities during a trial pilot, along with activities conducted in preparation for the trial. The trial preparation period was longer than anticipated due to contracting complexity, extensive health system technical security evaluations, and health system changes due to COVID-19 mitigation measures (e.g., temporary clinic shut-downs, staffing losses, and switch from in-person to virtual care).

### Target patient population

Participating clinicians could prescribe reSET or reSET-O to any patient who they determined to be eligible based on the FDA label for each digital therapeutic product. For both products, the label requires patients to be ≥ 18 years old, have access to a smartphone device or tablet, speak the English language, and can consent the vendor’s terms of service and privacy policy. reSET required a diagnosis of SUD, and reSET-O required an active buprenorphine prescription for the treatment of OUD [34]. To characterize the patient population of the participating clinics, the descriptive analysis sample included patients who had a primary care visit in the period from 2 weeks before through the end of the trial active implementation period and were 18 years or older at the time of the visit. Patients were attributed to the clinic where their qualifying primary care visit occurred.

### Data sources

Implementation costs were calculated using primary data collection of activities related to the rollout of reSET and reSET-O and included two sets of data. First, we developed structured meeting activity logs, adapted from prior research [40,41], that captured individual attendance and duration of regular meetings attended by multiple implementation participants. Information was obtained from meeting notes, agendas, calendar invites, and attendance reports and entered into the activity log by a study Research Specialist. Second, time use surveys were distributed to personnel involved in the implementation to capture time devoted to activities outside of regular meetings (see Glass et al. (2023) [Supplemental Material] for examples) [34]. For study team members, surveys were administered monthly. For clinical leaders, surveys were administered approximately twice monthly to coincide with a regular operations meeting to ensure completion. In addition, to minimize reporting burden, IMH specialists involved in the trial were randomly sampled on a weekly basis to complete a survey once during the implementation period. IMH specialists were contacted through institutional email, sent on behalf of the IMH leadership. Up to three follow-up reminders were sent to IMH specialists. We linked activity logs and survey data to public data on average salaries by occupation from the Bureau of Labor Statistics. Data tracking implementation activities was collected using these processes during both the pre-trial and active trial periods.

Intervention costs were calculated using administrative data routinely collected by the health system. This includes Clarity® databases for EHR data and Cost Management Database (CMD). EHR and claims data capture visit characteristics such as patient, provider type, visit type, visit department, procedures, ICD code sets, and lab, pharmacy, enrollment and patient sociodemographic data. These databases specifically include primary care clinic, clinician, prescriptions, and screening and assessment results. Claims databases cover contracted care such as emergency, hospital, and specialty addiction treatment visits. The CMD is the health system’s costing system that allocates direct and indirect costs to all services. CMD processes and reports costs incurred to provide medical services to enrollees. These costs include direct patient care costs (direct costs) and administrative costs (indirect costs) related to providing patient care services.

### Implementation cost calculations

We conducted descriptive analysis reporting time and associated costs of implementing reSET and reSET-O. To accomplish this goal, we aggregated micro-level activity records capturing time usage recorded in structured meeting activity logs and time use surveys. Time devoted to implementation activities and associated opportunity costs were reported across several dimensions including trial arm, implementation activity category, participant role and month of trial. Implementation activity categories included 1) leadership activities ensuring organizational support, such as communicating with organizational leaders and obtaining health system policy approvals); 2) training development and preparation, 3) EHR tools development and revisions, 4) activities to support health coaching, 5) performance reporting activities to communicate standard implementation progress to health system leaders, 6) standard implementation activities in the clinics related to the ongoing work by and in supervision of reSET-trained clinicians, 7) general implementation preparation (e.g., contracting agreements, tech risk approvals, reference material development, meeting scheduling and preparation, project management), 8) practice facilitation, and 9) formative evaluation. Time in all categories specifically captured activities to increase adoption of the digital therapeutic, and not activities solely for research. Participants recorded estimates of time spent in each of these categories on time use surveys, and meeting topics were recorded in meeting activity logs and mapped onto these categories.

Implementation activities reported by IMH specialists included interactions with patients about reSET or reSET-O, reviewing training materials and documents, using the vendor’s clinical dashboard, discussions with colleagues and implementation team members, reviewing reports, and other activities related to reSET or reSET-O. All activities by IMH specialists were considered part of cost category (6), ongoing work by reSET-trained clinicians.

To account for IMH specialists being randomly sampled to complete the time surveys over the trial period, we applied regression models to extrapolate activity time across all IMH specialists. This was completed by estimating a linear regression model with hours devoted to implementation as the outcome and including month of implementation, site fixed effects and intervention arm as predictors. We then used the method of recycled predictions [42] to extrapolate activity time estimates for the population of all IMH specialists for each month of the implementation.

Then, to monetize time spent on implementation activities, we multiplied participation time (converted to hours) by the opportunity cost of time, measured as participants’ hourly wage rates. Hourly wages were estimates reflecting the average wage for a participant’s occupation in the metropolitan area of employment [43]. To account for inflation, we adjusted wages to 2023 constant dollars using the Personal Health Care Index [44,45]. We adjusted to 2023 dollars given this was the last calendar year of the trial.

Implementation costs also included direct expenditures on resources to support implementation (e.g., operating costs). Operating costs are dollars spent on regular operation of reSET and reSET-O incurred by the delivery system. Costs include infrastructure/technical resources and staff support such as IT support, digital therapeutic prescription costs, and capital equipment. We calculated space costs by multiplying staff full time equivalent (FTE) spent on implementation activities by office space costs per FTE in Seattle, WA. Also included EHR programmer and health coach FTE in operating costs. Both positions were paid for by the research grant, but the health coach salary cost-transferred to the care delivery system so the position could be hired, credentialed, and supervised by the mental health service line.

### Clinical intervention costs

We measured the intervention costs from a health system perspective associated with providing reSET and reSET-O. Intervention costs were captured for all patients who were prescribed reSET or reSET-O, and were attributed to the clinic where the therapeutic was prescribed. We included costs of three types of outpatient visits with an ICD-10 diagnosis of SUD (F1*.*, except F17.*). First, we included the costs of the outpatient visit with a IMH specialist or primary care provider when reSET or reSET-O was prescribed. Second, because patients must subsequently activate the prescription prior to use, we also included the cost of one outpatient visit after the prescription date, but before the activation date for patients who received a prescription. This accounted for a potential visit to address any questions or issues patients may have in activating the digital therapeutic. For patients with multiple visits before the activation date, we calculated cost using only the first visit following their prescription date. For patients who did not activate the digital therapeutic, we included the cost of the earliest visit within 30 days after the prescription date, because job aids suggested clinicians discuss activation at a subsequent visit. Third, for patients who activated the prescription, we included SUD related encounters between the activation date and the following 12 weeks, the duration of the reSET or reSET-O prescription. Cost for each of these encounters comprised of both direct (e.g., cost of labor) and indirect (e.g., overhead costs) costs related to providing patient care services. The indirect component includes costs incurred in providing patient care that were not directly related to individual patient services. Examples include expenses for care delivery facilities, clinic administration and malpractice insurance. Prescription costs for reSET or reSET-O were not included because the digital therapeutic was provided by the vendor for a single licensing fee, which was incorporated into operating costs. Historically, the wholesale acquisition cost of reSET and reSET-O was $1,665 [46,47].

### Analysis

We first present descriptive statistics of patients who had a primary care clinic visit prior to the active implementation period. Patient characteristics were constructed using data in the prior two years. We conducted descriptive analysis reporting time and associated cost of implementing reSET or reSET-O, which include estimates of means and standard deviations across sites. Implementation costs were reported by trial arm, implementation activity type, participant role and time. We conducted analogous descriptive analyses of intervention costs. All analyses were conducted using RStudio, (Posit Software, Boston, MA).

### Ethics statement

The Kaiser Permanente Interregional Institutional Review Board granted ethical approvals. The analyses described in this report were approved under IRB record numbers 1743121 and 1794767. Waivers of consent were granted to randomize clinics, and to access, use and collect data because the study was deemed to have no more than minimal risks and it would not be otherwise feasible to conduct the study.

## Results

### Characteristics of target patient population

Table 1 presents summary statistics of all patients who visited primary care clinics, stratified by trial arm. We identified 51329, 69295, 76696 and 60894 patients for the standard implementation, practice facilitation, health coaching and practice facilitation plus health coaching groups, respectively. Among all patients across arms, average age was 52.6 years (SD = 18.6) and the majority resided in urban areas (97.2%) and were enrolled in the health system for at least a year (81.0%). A notable proportion had a mental health diagnosis (31.8%), including anxiety disorder (24.7%). Across all insurance types, the modal category was commercial (39.3%).

**Table 1:** Demographic and clinical characteristics of primary care patients across trial arms.

	Trial Arm				
	SI (N = 51329)^1^ N (%)	PF (N = 69295) N (%)	HC (N = 76696) N (%)	PF + HC (N = 60894) N (%)	Total (N = 258214) N (%)
**Age in years**	51.8 (18.2)	55.9 (18.5)	51.3 (18.7)	51.1 (18.2)	52.6 (18.6)
**Age in years(categories)**					
18-24	3697 (7.2)	4299 (6.2)	5909 (7.7)	5016 (8.2)	18921 (7.3)
25-34	7849 (15.3)	7851 (11.3)	13420 (17.5)	9201 (15.1)	38321 (14.8)
35-44	8178 (15.9)	8824 (12.7)	11919 (15.5)	9930 (16.3)	38851 (15.0)
45-54	8347 (16.3)	9334 (13.5)	11307 (14.7)	9948 (16.3)	38936 (15.1)
55+	23258 (45.3)	38987 (56.3)	34141 (44.5)	26799 (44.0)	123185 (47.7)
**Gender**					
Male	20023 (39.0)	28383 (41.0)	31317 (40.8)	24554 (40.3)	104277 (40.4)
Unknown	0 (0.0)	0 (0.0)	0 (0.0)	2 (0.0)	2 (0.0)
**Race**					
Hispanic	3158 (6.2)	3576 (5.2)	5536 (7.2)	4201 (6.9)	16471 (6.4)
Non-Hispanic					
Asian	6803 (13.3)	2875 (4.1)	8176 (10.7)	4988 (8.2)	22842 (8.8)
Black or African American	4105 (8.0)	1391 (2.0)	4454 (5.8)	2697 (4.4)	12647 (4.9)
American Indian or Alaska Native	227 (0.4)	460 (0.7)	314 (0.4)	341 (0.6)	1342 (0.5)
Native Hawaiian or Pacific Islander	526 (1.0)	547 (0.8)	598 (0.8)	622 (1.0)	2293 (0.9)
White	29719 (57.9)	53756 (77.6)	47929 (62.5)	40187 (66.0)	171591 (66.5)
Multiple Race	1599 (3.1)	1829 (2.6)	2005 (2.6)	1549 (2.5)	6982 (2.7)
Other	965 (1.9)	731 (1.1)	1336 (1.7)	916 (1.5)	3948 (1.5)
Unknown	4227 (8.2)	4130 (6.0)	6348 (8.3)	5393 (8.9)	20098 (7.8)
**Rurality of Residence**					
Urban	50836 (99.0)	64531 (93.4)	75601 (98.6)	59924 (98.4)	250892 (97.2)
Missing values	196 (0.38)	240 (0.35)	499 (0.65)	414 (0.68)	1349 (0.52)
**Insurance Type**					
Medicaid	2005 (3.9)	2517 (3.6)	2145 (2.8)	2131 (3.5)	8798 (3.4)
Medicare	13239 (25.8)	25775 (37.2)	19951 (26.0)	15231 (25.0)	74196 (28.7)
State subsidized	2010 (3.9)	1905 (2.7)	3416 (4.5)	2415 (4.0)	9746 (3.8)
Private Pay, Self-funded, High Deductible and Basic Health	10465 (20.4)	8435 (12.2)	16716 (21.8)	12584 (20.7)	48200 (18.7)
Commercial	20624 (40.2)	25736 (37.1)	29709 (38.7)	25384 (41.7)	101453 (39.3)
Unknown	2986 (5.8)	4927 (7.1)	4759 (6.2)	3149 (5.2)	15821 (6.1)
**At least 1 year of enrollment**	41369 (80.6)	56997 (82.3)	61034 (79.6)	49741 (81.7)	209141 (81.0)
**Health status**					
Mental health diagnosis	15810 (30.8)	22611 (32.6)	24486 (31.9)	19103 (31.4)	82010 (31.8)
Anxiety	12464 (24.3)	17024 (24.6)	19320 (25.2)	15030 (24.7)	63838 (24.7)
Depression	8849 (17.2)	13204 (19.1)	13668 (17.8)	10831 (17.8)	46552 (18.0)
Serious Mental Illness	1237 (2.4)	1703 (2.5)	1760 (2.3)	1289 (2.1)	5989 (2.3)
**Positive screen for depression**					
Yes	7249 (14.1)	9881 (14.3)	10848 (14.1)	8852 (14.5)	36830 (14.3)
Missing	14713 (28.7)	18314 (26.4)	22080 (28.8)	17571 (28.9)	72678 (28.1)
**Any emergency visits**	182 (0.4)	309 (0.4)	309 (0.4)	285 (0.5)	1085 (0.4)
**Any hospitalization**	274 (0.5)	446 (0.6)	408 (0.5)	367 (0.6)	1495 (0.6)
**SUD-related characteristics**					
**High-scoring drug use screens**					
Alcohol (Yes)	1037 (2.0)	1345 (1.9)	1729 (2.3)	1284 (2.1)	5395 (2.1)
Alcohol (Missing)	11875 (23.1)	15154 (21.9)	17597 (22.9)	14737 (24.2)	59363 (23.0)
Cannabis (Yes)	2518 (4.9)	3449 (5.0)	3982 (5.2)	2955 (4.9)	12904 (5.0)
Cannabis (Missing)	11959 (23.3)	15274 (22.0)	17701 (23.1)	14835 (24.4)	59769 (23.1)
Other Drug (Yes)	848 (1.7)	856 (1.2)	1777 (2.3)	855 (1.4)	4336 (1.7)
Other Drug (Missing)	12098 (23.6)	15449 (22.3)	17817 (23.2)	14938 (24.5)	60302 (23.4)
**SUD Diagnosis**					
Alcohol	875 (1.7)	1401 (2.0)	1449 (1.9)	1205 (2.0)	4930 (1.9)
Cannabis	290 (0.6)	398 (0.6)	432 (0.6)	310 (0.5)	1430 (0.6)
Opioid	359 (0.7)	520 (0.8)	502 (0.7)	418 (0.7)	1799 (0.7)
Stimulant	117 (0.2)	129 (0.2)	161 (0.2)	139 (0.2)	546 (0.2)
Other Drug	172 (0.3)	220 (0.3)	217 (0.3)	176 (0.3)	785 (0.3)
Any drug overdose	50 (0.1)	69 (0.1)	71 (0.1)	55 (0.1)	245 (0.1)
**Prescriptions buprenorphine**	381 (0.7)	522 (0.8)	638 (0.8)	469 (0.8)	2010 (0.8)
**Engagement in SUD-related care**					
Mental health specialty	1132 (2.2)	2090 (3.0)	2119 (2.8)	1457 (2.4)	6798 (2.6)
Addictions	28 (0.1)	11 (0.0)	33 (0.0)	41 (0.1)	113 (0.0)
Social work	46 (0.1)	39 (0.1)	42 (0.1)	56 (0.1)	183 (0.1)
None	50123 (97.7)	67155 (96.9)	74502 (97.1)	59340 (97.4)	251120 (97.3)

SI = Standard Implementation, PF = Practice Facilitation, HC = Health Coaching, SUD = substance use disorder

Sites in all trial arms received standard implementation.

^1^Sample sizes include all eligible primary care patients at included sites.

Characteristics were similar across trial arms in several characteristics including male gender (39.0% to 41.0%), diagnosis of any mental health condition (30.8% to 32.6%), diagnosis of stimulant use disorder (0.2%) or OUD (0.7% to 0.8%), record of buprenorphine medication prescription (0.7% to 0.8%) and visited the ED (0.4% to 0.5%). Compared to other trial arms, patients in the practice facilitation arm were slightly older (55.9 years, SD = 18.5), more likely to be of white race (77.6%) and more likely to be covered by Medicare (37.2%). Across all trial arms, patients who had received any SUD treatment rarely did through Addiction Medicine (0.0% to 0.1%) or Social Work (0.1%).

### Implementation costs by study arm

Implementation costs included direct expenditures on resources to support implementation and operating costs to support regular operation of reSET and reSET-O incurred by the delivery system. Total implementation costs over the 50-month study period were $748,088. Implementation ($510,709) and operating ($237,379) costs represented 68.3% and 31.7% of total costs, respectively (Table 2). Operating costs stemmed from staffing an EHR programmer and health coach, and cost of office space. Of the four study arms, standard implementation with practice facilitation and health coaching was the costliest approach, accumulating $288,173 over the study period. Standard implementation with health coaching was the next highest in costs ($182,718) followed by standard implementation with practice facilitation ($153,327) and standard implementation alone ($123,871). Dividing these costs by the corresponding number of study sites in each arm indicate the standard implementation, standard implementation with practice facilitation, standard implementation with health coaching, and standard implementation with practice facilitation and health coaching was $24,774, $30,665, $36,544 and $48,029 per site, respectively.

**Table 2:** Implementation costs by trial arm (in 2023 US dollars).

	Trial Arm				Total
	Standard	PF	HC	PF + HC	
Cost Category					
Personnel Time	79,372	129,851	109,006	192,480	510,709
Operating	44,498	23,477	73,711	95,693	237,379
Total	123,871	153,327	182,718	288,173	748,088
# Primary Care Sites	5	5	5	6	21
Cost Per Site	24,774	30,665	36,544	48,029	35,623

PF = Practice facilitation, HC = Health Coaching.

### Implementation costs by activity

Activities in general implementation preparation were the costliest component, which encompassed 1,821 hours of staff time, translating to $108,462 in costs (Table 3). However, activities devoted to practice facilitation represented a greater number of hours (2,019) translating to $105,499 in costs. Three other categories accumulated over 1,000 hours of activity time: formative evaluation (1,694 hours, $71,256), standard implementation activities in clinics (1,664 hours, $88,320), and health coaching and protocol management (1,043 hours, $60,153). Three categories represented a relatively smaller portion of implementation activities: EHR tools development (239 hours, $14,868), ensuring organizational support (201 hours, $15,510) and performance reporting (130 hours, $10,725).

**Table 3:** Summary of implementation costs by activity category.

Activity Category	Total Hours	Cost ($)^1^
Standard Implementation	1,664.2	88,320
Practice Facilitation	2,018.8	105,499
Formative Evaluation	1,693.7	71,256
General implementation preparation	1,821.1	108,462
Health Coaching Protocol Management and Development	1,043.4	60,153
Training	566.6	35,917
EHR Tool Development	239.0	14,868
Ensuring organizational support	201.0	15,510
Performance Reporting	129.7	10,725
**TOTAL**	**9,377.5**	**510,709**

^1^Activity categories sum to more than the total due to rounding.

### Implementation costs by role

The Project Manager and Practice Facilitators devoted the most time to implementation activities, representing 2,364 hours (time cost = $142,452) and 2,057 hours (time cost = $109,371), respectively (Table 4). A notably high level of effort, representing over 800 aggregate hours, was devoted by the Principal Investigator (902 hours, $76,438), Formative Evaluators (881 hours, $46,817) and Economic Evaluators (829 hours, $19,894). A third group of personnel devoted a slightly lower level of time to implementation, representing approximately 500 hours during the trial. These included Programmers/IT Staff (522 hours, $45,466), Delivery System Leaders (501 hours, $35,006) and IMH Specialists (488 hours, $19,339).

**Table 4:** Summary of implementation costs by study role.

Role	Total Hours	Cost ($)
Co-Investigator	39.50	3,346.49
Delivery System Leaders	501.00	35,005.83
Economic Evaluators	828.92	19,894.00
Formative Evaluators	880.62	46,816.63
Health Coaches	122.47	2,992.19
Other	3.00	170.56
Practice Facilitators	2,057.26	109,371.06
Principal Investigator	902.23	76,438.25
Programmers/IT Staff	521.88	45,466.32
Project Managers	2,364.17	142,452.41
Research Specialists	389.92	9,416.33
IMH Specialists	487.94	19,339.38
Unknown	278.50	0.00
**TOTAL**		**510,709.45**

IMH = Integrated Mental Health.

$0 cost for unknown category results from the inability to identify specific staff members who conducted 278.5 hours to implementation.

### Implementation cost over time

**Fig 1:**
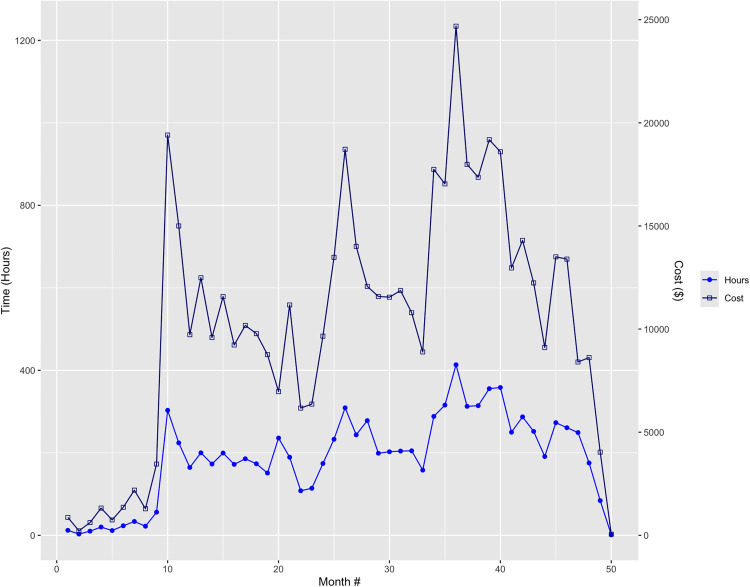
Implementation costs by study month.

Total implementation costs exhibited variation across the study period, with several notable trends (Fig 1). Between months 1 and 7, costs per month increased from $867 to $2,188 as study activities slowly ramped up. Costs rapidly accelerated in months 9 ($3,455) and 10 ($19,410) and remained elevated in month 11 ($15,001) because the team engaged in intensive EHR tool design efforts. Monthly costs then gradually declined from $12,490 in month 12 to $6,168 in month 22. Costs then accelerated from $6,361 in month 23 to $18,719 in month 28, representing the first three months of the standard implementation pilot and included activities such as training and orientation sessions. Costs then declined from $14,013 to $8,891 between months 28 and 33 as the implementation team piloted the health coaching and practice facilitation strategies and conducted final preparation for trial launch. Costs sharply increased to $17,743 in month 34, followed by a subsequent increase to $24,687 in month 36, corresponding to the start of the implementation trial. Costs then exhibited a gradual decline to $9,109 in month 44. Costs temporarily increased to approximately $13,500 in month 45 and 46. In the proceeding months, costs subsequently declined to $53 in the last month of the implementation trial.

### Intervention costs

Intervention costs were highest in the standard implementation with practice facilitation and health coaching with 26 patients prescribed reSET who incurred $18,051 in costs (Table 5). The second highest arm was standard implementation with practice facilitation with 26 patients incurring $11,492 in costs. Costs were markedly lower in the standard implementation with health coaching (3 patients incurring $967) and standard implementation alone (6 patients incurring $1,879) arms.

**Table 5:** Summary of intervention cost by study arm.

Arm	Total Cost ($)	# Patients Prescribed reSET
Standard Implementation alone	1,878.92	6
Standard Implementation with Practice Facilitation	11,492.12	26
Standard Implementation with Health Coaching	966.73	3
Standard Implementation with Practice Facilitation and Health Coaching	18,051.18	26

## Discussion

Strategic implementation approaches, successfully utilized by health systems to increase uptake of treatments for behavioral health conditions, offer a potential solution to the limited accessibility of digital therapeutics for SUDs. In addition to quantifying the clinical benefits of these implementation strategies, health systems also require insights informing their value proposition. To address this priority, this study quantified the implementation and intervention costs associated with four strategies to increase reach and adoption of FDA-authorized digital therapeutics for SUD, reSET and reSET-O, during the preparation and conduct of an implementation trial. Economic evaluation such as this study can influence the acceptability, adoption and sustainability of an evidence-based practice [48].

Another key innovation of this study is the decomposition of implementation costs by activity category and implementation role. Activities related to implementation preparation and execution of standard implementation each represented a notably large share of time and associated costs. Activities related to general implementation preparation also reflected substantial staff resources. Costs in two categories were $88,320 and $108,426, representing 17% and 21% of the $510,709 total in personnel costs. Together, staff costs in these categories reflect the substantial effort required for setting up an implementation trial of digital therapeutics, including activities such as obtaining health system approvals and establishing contractual agreements with the developer of the digital therapeutic, coordination with individual clinics and trainings with clinical staff, and preparing for and conducting facilitation meetings in the clinics. The high cost of standard implementation reflects regular meetings with implementation staff over the duration of the trial. Also of note is the fact that implementation pivoted to a virtual modality at the start of the COVID-19 pandemic. Substantial time was devoted to developing processes to execute implementation activities and setting up workflows for prescribing reSET or reSET-O virtually, which was not expected at the outset of the study.

Two additional activity categories, practice facilitation ($105,499) and formative evaluation ($71,256), were notable for their markedly high costs. These implementation categories were particularly labor intensive, reflecting the substantial effort required to plan for and execute activities. For example, practice facilitation involved monthly meetings with front-line staff, and ad-hoc support between meetings. Formative evaluation involved multiple cycles of qualitative observations, interviews, and analysis, followed by the preparation and delivery of regular presentations to health system partners to inform implementation decision making, including adaptations. While formative evaluation was important to improve the implementation, health systems may choose to scale back or forgo their own rigorous formative evaluation.

Study findings demonstrate considerable variation in implementation hours and costs by role. This wide variation reflects the diverse expertise required to execute activities across several distinct implementation strategies. Notably elevated costs among the project manager and principal investigator, the former reflecting the highest cost group, indicate their all-encompassing role in the implementation effort. This includes interfacing with personnel across all aspects of the project and across each of the study arms. Practice facilitators were the group with the second highest costs, embodying their contributions to preparing and executing practice facilitation with sites, and their participation in meetings that were part of standard implementation. Estimated hours among IMH specialists totaled over 500 hours, reflecting their presence across 21 sites participating in the trial. IMH specialists participated in a training at the start of the trial and conducted activities to enable delivery of reSET and reSET-O to patients, including time reading manuals and training materials, reviewing EHR dashboards and communicating with patients about the digital therapeutic. These activities performed by IMH specialists were considered part of standard implementation. The relatively high implementation costs among formative evaluators reflect the labor-intensive nature of these activities, as previously discussed. Note formative evaluators and economic evaluators conducted implementation activities to provide guidance to decision-makers about the adoption of digital therapeutics more broadly across the health system.

Comparison of costs by study arm revealed the costliest implementation approach being the combination of standard implementation, practice facilitation, and health coaching. This reflects sites being exposed to the most intensive implementation approach involving activities from all three distinct strategies. Standard implementation plus health coaching was next costliest, reflecting a full-time salary for a certified health coach whose dedicated job was to support patients prescribed reSET and reSET-O.

Intervention costs in each trial arm were substantially lower than corresponding implementation costs. The low intervention costs were due to the limited reach of the digital therapeutic, as described in other studies [49]. Low patient reach underscores several implementation challenges experienced during the trial, such as staff shortages of IMH specialists and difficulties engaging patients through a virtual modality.

This study contributes to a nascent literature on the economic impacts of implementing evidence-based approaches for mental health and SUDs [33,40,50–52]. To our knowledge, there is no direct study to compare our results against. However, several studies have examined the economics of practice facilitation strategies in the substance use and behavioral health clinical areas. Garcia et al. (2023) examined an external facilitation approach to increase access to medication treatment for OUD, finding sites incurred an average of $18,847 in implementation costs [51]. Yeung et al. (2020) examined the cost of implementing behavioral health integration in a large primary care system, revealing that clinics accrued $63,486 in costs [52]. Wong et al. (2022) examined external facilitation to implement trauma-focused psychotherapy for post-traumatic stress disorder, finding costs averaged $21,482 per implementation site [40]. Cost per site across trial arms from $19,390 to $46,652 in this study are within the range of estimates in literature. Cost estimates in our study and prior work should account for the intensity and duration of practice facilitation strategy, number of involved clinics, the size of the target patient population as well as the detailed components of implementation costs included in the analysis.

This study has several limitations. First, measurement of implementation costs relied on primary data collection from staff involved in implementation. We took intentional steps to minimize respondent burden and maximize response rates. Nevertheless, staff-reported data may be incompletely measured and subject to recall bias. Second, data collection captured time devoted to implementation activities and categories where effort was devoted. Data on intensity or quality of effort, which is substantially more difficult to ascertain, was not collected. Third, implementation overlapped with the COVID-19 pandemic, which resulted in several unique circumstances, such as transitioning implementation activities to a virtual modality and project delays prior to launching the pilot phase. Interpretation of study findings should account for this and other pandemic-specific phenomenon. Fourth, the scope of our descriptive approach sought to describe implementation costs across several dimensions, but did not examine drivers of site variation in implementation costs among sites. This was primarily because a large proportion of costs were fixed in nature and incurred to launch the strategies across all sites. Finally, the implementation strategies examined in this study were focused on increasing uptake of an evidence-based treatment for SUDs. While many elements of the strategies are likely applicable to implementing evidence-based treatments for other medical conditions, future studies should examine economic impacts in these settings directly.

In summary, this study addresses the substantial gap in scientific evidence quantifying the economic impacts of implementation strategies to increase uptake of evidence-based treatments or SUDs. Findings from this study are relevant to health systems seeking to understand the economic investments required to employ implementation strategies shown to increase uptake of evidence-based practices for behavioral health conditions and SUDs [53–57]. Specifically, estimates in this study provide a frame of reference for health systems on the expected costs of adopting theoretically rooted strategies that have been demonstrated to be successful. Correspondingly, these estimates can help inform the design and allocation of resources to population level approaches addressing SUDs.
